# A positive effect of Cerebrolysin on motor functions and spasticity in ALS with limb or bulbar onset is questionable

**DOI:** 10.25122/jml-2024-0158

**Published:** 2024-02

**Authors:** Josef Finsterer

**Affiliations:** 1 Neurology Department, Neurology & Neurophysiology Center, Vienna, Austria

We read with interest the article by Firstenfeld et al. [1] investigating the effects of Cerebrolysin in amyotrophic lateral sclerosis (ALS) using a prospective, single-center, randomized, double-blind, placebo-controlled trial. Patients (*n* = 20) with limb-onset or bulbar-onset ALS received Cerebrolysin (10 ml/day) for 5 days per week in the first month, followed by 3 days per week in months two and three [1]. Patients receiving Cerebrolysin experienced a 2.3-fold increase in the ALS functional rating scale (ALSFRS) after 1 month, sustained over the following 2 months [1]. Secondary outcome measures (modified Ashworth scale, Beck’s depression inventory, walking tests, hand grip strength) also improved [1]. However, several limitations in the study design warrant discussion as they may influence the interpretation of the results.

One limitation is the absence of a cross-over phase. Including a cross-over design where patients in the control group receive Cerebrolysin after the initial three-month period would strengthen the evidence for the effectiveness of Cerebrolysin. Assuming that Cerebrolysin was useful, one could also expect a positive effect in the 8 patients in the control group during the cross-over phase.

Furthermore, the study lacks detailed information on baseline patient characteristics. Specifically, the data on the distribution of disease subtypes (bulbar-onset vs limb-onset ALS) and ALS types (sporadic vs. familial) within both groups is not reported. This information is crucial as these factors can influence motor function and performance on outcome measures like walking tests.

Additionally, there are concerns regarding the interpretation of the walking test results. Walking time and distance walked within 2 min depend largely on the extent of lower limb impairment. Since ALS can begin with upper limb dominance, we should know whether the shorter walking time and the longer walking distance in the verum group could simply be due to the variable clinical presentation at admission, with less lower limb predominance in the verum group than the control group.

Another limitation is the relatively short follow-up period of 3 months. Since maintenance of functional level over a longer period is not uncommon in patients with ALS, depending on the stage of the disease, it is conceivable that maintenance of functional level simply represents the natural disease course and not a presumed treatment effect. It would have been desirable to follow the patients over a longer period. An observation period of 3 months is too short to assess the long-term outcome and effect of Cerebrolysin on motor functions and spasticity.

A further inconsistency appears in the results section. The inability to perform knee flexion analysis due to non-existent knee flexion in all patients seems to contradict the reported ability of all patients to perform walking tests. It should be explained how patients who could not bend their knees could walk. It should also be explained why not all patients could bend their knees. Was this due to fixed knee contractures or due to other causes?

Finally, the study does not specify comorbidities and co-medications besides riluzole. Knowledge of comorbidities and co-medications is crucial as they can greatly influence disease progression and outcomes in patients with ALS.

In conclusion, while the study by Firstenfeld et al. [1] offers valuable insights, the limitations discussed above complicate the interpretation of the results. Addressing these limitations could strengthen and reinforce the statement of the study. Based on the considerations expressed above, it is questionable whether the presumed beneficial effect of Cerebrolysin in patients with ALS is more likely due to patient selection, comorbidities, co-medications, and differential impairment of muscle groups between control and treatment patients.
